# Exploratory Data Analysis of Acceleration Signals to Select Light-Weight and Accurate Features for Real-Time Activity Recognition on Smartphones

**DOI:** 10.3390/s131013099

**Published:** 2013-09-27

**Authors:** Adil Mehmood Khan, Muhammad Hameed Siddiqi, Seok-Won Lee

**Affiliations:** 1 Division of Information and Computer Engineering, Ajou University, San 5 Woncheon-dong, Suwon 443-749, Korea; E-Mail: amtareen@ajou.ac.kr; 2 Department of Computer Engineering, Kyung Hee University, Suwon 446-701, Korea; E-Mail: siddiqi@oslab.khu.ac.kr

**Keywords:** accelerometer sensor, smartphone, context-awareness, activity recognition, expolatory data analysis, feature extraction

## Abstract

Smartphone-based activity recognition (SP-AR) recognizes users' activities using the embedded accelerometer sensor. Only a small number of previous works can be classified as online systems, *i.e.*, the whole process (pre-processing, feature extraction, and classification) is performed on the device. Most of these online systems use either a high sampling rate (SR) or long data-window (DW) to achieve high accuracy, resulting in short battery life or delayed system response, respectively. This paper introduces a real-time/online SP-AR system that solves this problem. Exploratory data analysis was performed on acceleration signals of 6 activities, collected from 30 subjects, to show that these signals are generated by an autoregressive (AR) process, and an accurate AR-model in this case can be built using a low SR (20 Hz) and a small DW (3 s). The high within class variance resulting from placing the phone at different positions was reduced using kernel discriminant analysis to achieve position-independent recognition. Neural networks were used as classifiers. Unlike previous works, true subject-independent evaluation was performed, where 10 new subjects evaluated the system at their homes for 1 week. The results show that our features outperformed three commonly used features by 40% in terms of accuracy for the given SR and DW.

## 1. Introduction

Context-awareness is an essential part of ubiquitous computing, and human activity recognition (HAR) has emerged as an important tool to identify the user's context for automatic service delivery in ubiquitous application. For example, in the case of ubiquitous healthcare applications, recognition of everyday activities could enable such systems to watch and learn any changes in daily behavior of an elderly person that might be the indicators of developing physical or mental medical conditions.

The first step towards achieving the goal of recognizing the activities of daily living is to equip HAR systems with sensing capabilities. Two main approaches have been employed for this purpose: external (in which devices are fixed to predetermined points of interest) and wearable sensors (in which devices are attached to the user). Smart-homes equipped with sensors embedded in everyday objects [1–3] and HAR by means of video cameras [4,5] fall in the category of external sensors. The main problem with an external approach is its lack of pervasiveness, *i.e.*, it forces the user to stay within a perimeter defined by the position and capabilities of the sensors.

As for a wearable approach, a range of wearable sensors has been used to capture and analyze human movement in free-living subjects [6]. Of these sensors, accelerometers are becoming widely accepted as useful tools for the assessment of human motion in clinical settings and free-living environments [6].

In spite of the fact that accelerometery has emerged as an inexpensive and reliable means of HAR, the majority of accelerometer-based HAR systems developed have used a separate sensor device attached to subjects' bodies [6–18]. However, this solution is obtrusive and very few people would like to have sensors attached to their bodies, or wear special t-shirts, bracelets or belts for that purpose.

These days, smartphones come equipped with a variety of sensors, including accelerometers. These devices are part of people's daily lives. People carry smartphones nearly everywhere they go. Consequently, such devices can be employed in creating valid and reliable measures of physical activity continuously over longer periods of time during free-living conditions. Recently, many studies have incorporated accelerometer-enabled smartphones for the sake of HAR, such as [19–29]. However, most of these works have employed smartphones as mere data collection devices, which sent data to an accompanying device (such as PC) for further processing.

Feature extraction plays a vital role in any accelerometer-based HAR system. Since mobile phones are generally energy constrained and extending phone battery life is an essential requirement, using smartphones for HAR thus requires features that are both light-weight (energy efficient) and accurate (possess high discriminating power) to preserve battery life and ensure high accuracy. A large number of frequency and time domain features has been investigated in the past with varying success rates. The most widely used time domain features include: mean [8–10], variance or standard deviation [8,10], energy [8–10], entropy [9], correlation between axes [8–10], signal magnitude area [11], tilt angle [11], autoregressive (AR) coefficients [12], and so on. The most popular frequency domain features used so far are the Fast Fourier Transform (FFT) [13,30,31] and Discrete Cosine Transform (DCT) coefficients [32].

Frequency domain features require higher components to discriminate between different activities. Their calculation requires longer time windows, and thus they increase computational cost and are not suitable for real-time applications. On the other hand, time domain features can be easily extracted in real-time. Therefore, they are popular in many practical accelerometer-based HAR systems. Although activity recognition using time domain features was successful to some extent, the recognition results using these features have not had a high success rate because such methods assume that activity acceleration signals are deterministic. Therefore, it is still desireable to investigate what could be the best features for real-time smartphone-based HAR.

In our previous work in this field, we proposed an artificial neural network based hierarchical classification scheme that used a mix of different features to classify 15 activities with a high accuracy [33]. The data in this system were collected using a commercial accelerometer device attached to a subject's chest. Though the system provided high accuracy, it was an off-line system in the sense that the data were transferred to a computer by means of a bluetooth where further processing was carried out. Moreover, it was not a truly subject-independent system as the same subjects took part in both training and testing.

Accordingly, the contributions of this research are fourfold. Firstly, this work implements an online HAR-system in which the whole recognition process (preprocessing, feature extraction and classification) is done on a smartphone with a built-in accelerometer. Secondly, this study performs exploratory data analysis of the 3-axes acceleration signals captured from the phone to find features that are not only robust in representing these signals across multiple subjects but could also be computed in real-time using small time windows and low sampling rate. Thirdly, it allows users to carry their phones freely at 3 different positions. Lastly, the system was evaluated in a true subject-independent method, using subjects that were not part of the training process, and at different sampling rates to find the most accurate and light-weight features.

The rest of the paper is organized as follows. Section 2 discusses some related work. Section 3 explains the way this research study was carried out. Section 4 describes the data collection scheme. Section 5 explains the algorithms that are used at different stages of our system. Section 6 summarizes the implementation details, and experimental results. Section 7 provides some discussion, whereas Section 8 concludes the paper.

## 2. Related Work

HAR-using mobile phones with embedded accelerometers can be divided into two categories. The first case is where the data is collected using a mobile phone and the activity recognition is done afterwards on a PC/server [19–21,23–26,34,35]. The second case (which comprises of very few studies) is where the complete activity recognition system runs purely on the smartphone (online systems) [22,27–29]. The focus of this paper is the latter case.

Among the above mentioned server-based approaches, [34,35] implemented real-time HAR systems on smartphones using the Thin-Client approach. Meaning, the raw acceleration data are sent to the server in real-time for feature extraction and classification. The classified labels are either sent back to the mobile device, or stored on the server for later use. A server is expected to have better processing, storage, and energy capabilities, which allows the use of more complex feature extraction and classification methods. However, running a HAR system solely on a mobile device brings important benefits. For example, it reduces the energy expenditures, as the system does not require the raw data to be continuously transmitted to the server for processing. Such a system is more robust and responsive, as it does not depend on unreliable wireless communication links that may be unavailable or error-prone. This is a very important requirement for systems that require real-time decision making, such as medical or military applications. Finally, a mobile HAR system is more scalable, as performing the feature extraction and classification computations locally on the mobile device alleviates the server load.

In [28], Frank *et al.* introduced a system for activity and gait recognition for smartphones. The presented system is capable of performing the classification in real-time and can be trained on a smartphone. For their recognition system, the authors used geometric template matching as the feature extraction technique and support vector machines as the classifier. Though the paper claims that the presented system is capable of building new models and performing classification in real-time without drastically reducing phones battery life, the article does not include crucial information such as sampling frequency and classification rates, which makes it difficult to evaluate their system.

Kwapisz *et al.* [22] introduced a system that uses phone-based accelerometers to recognize six activities with a very high accuracy. They used a total of 43 features. The data were collected from 29 subjects using a custom-build Android application. During data collection, subjects carried their phones in their pants leg pockets. Each phone was configured to provide acceleration data at a sampling frequency of 20 Hz. However, given a small sampling frequency, the authors had to select a longer data-window (10 s) to obtain a high accuracy. Moreover, same subjects were used for both training and testing the system.

In [29], the authors have presented a personalized mobile activity recognition system for smartphones. Their system is capable of building and continuously updating the classification model on-board the mobile device using data stream mining. However, just like [28], the paper has no mention of the overall accuracy and the authors did not provide any details about the kind of features and the sampling rates used in their work, which makes it difficult to evaluate their system.

Finally, in [27], a real-time mobile phone-based activity recognition system is introduced in which both the model building and the classification task is performed on the device. The system was trained using a total of 42 time domain features, and two classifiers (quadratic discriminant analysis and k-nearest neighbors) were compared to recognize 5 activities. In this experiment, phones were configured to provide raw acceleration data at a rate of 40 Hz. Though the system achieved good accuracy in online experiments, it used a data-window of 7.5 s (slow response).

In conclusion, some excellent online approaches for smartphone-based activity recognition have been developed by researchers in the past; however, 4 problems can be identified. Firstly, some works have used high sampling rates whereas others have employed long data-windows to achieve a good accuracy. Secondly, features were selected without any formal analysis of the acceleration data. Thirdly, these works lacked experimental studies to see the effect of changing the sampling rate on the performance of these features. Lastly, most of the works have used the same subjects for both training and testing their systems, and have tried to limit the phone to a certain position. Accordingly, we have tried to resolve these issues in this work.

## 3. Methodology

This research work was carried out as following: (1) Activity acceleration data were collected from 30 subjects using 6 different sampling rates, and 3 different phone positions; (2) Exploratory data analysis was performed on this data to find features that are both lightweight and efficient to ensure long battery-life, fast response, and high recognition accuracy; (3) An accelerometer's output can vary for the same activity when carried in different positions, resulting in high within-class variance; therefore, to enable activity recognition for different positions, a method was needed after feature extraction that would suppress this variance. Several methods were studied for this purpose before selecting the Kernel discriminant analysis (KDA); (4) All the algorithms (feature extraction, KDA, and the classifier) were implemented in Java; (5) Classifiers were trained offline, and transferred to smartphones; (6) Lastly, real-time evaluations were performed on phones using 10 new subjects.

## 4. Data Collection

For this research work, activity data were collected from 30 healthy subjects (18 males and 12 females) between the ages of 26 and 35 years old, with an average height of 172.4 cm and average weight of 64 kg. Six common activities were selected: standing, walking, walking-upstairs, walking-downstairs, running, and hopping. The smartphone used in this data collection was an Android operating system based mobile phone called the LG Nexus 4. It is a smartphone from LG, equipped with a built-in triaxial accelerometer. A custom build application was used for data collection and annotation. The application had an added feature to adjust the sampling rate of the phone's accelerometer sensor prior to the data collection. Subjects were trained on the use of this application before data collection. Subjects were also requested to perform the activities in a natural way, without any fixed duration or seuqence. Each subject then collected the activity data at their homes in 6 sessions with a sampling rate of 20 Hz, 40 Hz, 60 Hz, 80 Hz, 100 Hz, and 120 Hz, respectively. 20 Hz was selected as the base case for model identification, validation and classification accuracy comparison. During the data collection, the subjects were told to place the smartphone in the following 3 positions: pants' left and right front pockets, pants' left and right back pockets, and jacket's inner pocket. They were instructed to collect roughly the same amount of data from each position. Initially the data were stored on the SD cards. Later they were moved to a computer for further analysis in MATLAB.

## 5. System Algorithms

### 5.1. Preprocessing

A 3-axes accelerometer embedded in a mobile phone carried by a user registers 2 kinds of acceleration along 3 dimensions (*x*, *y*, and *z*): a constant acceleration due to gravity, and any acceleration the mobile device is subjected to by the user. In order to calculate the real acceleration of the device, the effect of gravity must be eliminated. This was be done by applying a low-pass filter which builds a weighted average from all the history values. Thus, an abrupt peak will only push the mean value slowly. This helps in isolating the constant acceleration due to gravity which can then be simply subtracted from the sensor value to obtain the real acceleration of the device.

### 5.2. Exploratory Data Analysis (EDA) of Activity Data

EDA is a data analysis method that employs a variety of techniques (mostly graphical) to: maximize insight into a data set; uncover underlying structure; extract important variables; detect outliers and anomalies; test underlying assumptions; develop parsimonious models; and determine optimal factor settings [36]. Most EDA techniques are graphical in nature and are quite simple. These include scatter plots, lag plots, histograms, autocorrealtion plots, etc. These graphical tools are the shortest path to gaining insight into a dataset in terms of model selection, model validation, relationship identification and so on. In this work, the following EDA techniques were employed to find the model that can best describe activity acceleration signals:

- Autocorrelation plot: Autocorrelation plots are commonly used tools for identifying a model that can best describe a given time-series. Autocorrelation is the average of the product of a data sample with a version of itself advanced by a lag. The autocorrelation plot can provide answers to the following questions: (1) Is an observation related to adjacent observation? (2) Is the observed time series white noise? (3) Is the observed time series autoregressive? The autocorrelation function is described by the equation below,
  (1) $$r_{xx}[k]=\frac{1}{N}\sum_{n=1}^{N-k}x[n]x[n+k]$$
  where *r_xx_*[*k*] is the autocorrelation value of *x* at sample delay *k*, and *N* is the number of data points. For a very small advance, the values of the two signals at any given instant will be very similar. As the lag increases, the difference between the two values becomes larger. If a signal has both a periodic and a random component, the latter gradually disappears as the lag increases. This property is useful for extracting periodic signals from random noise [36].
- Lag plot: A lag plot performs the randomness check. In other words, it checks if a given time series or signal is random or not. Random data do not exhibit any structure whereas non-random data exhibit some sort of correlation in the lag plot [36]. A lag refers to a fixed time displacement. For example, given a time series *Y_1_, Y_2_*, …, *Y_N_*, a plot of lag 1 is a plot of the values of *Y_i_* versus *Y_i-_*_1_.

**Figure 1.**
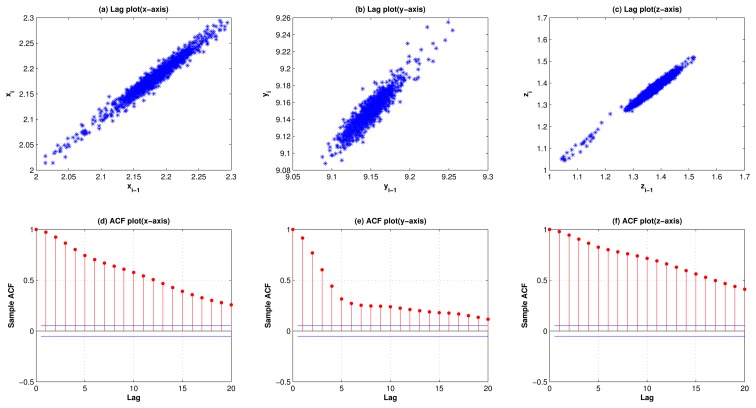
Lag plots (row 1) and autocorrelation plots (row 2) for the 3-axes activity-acceleration signals of standing, showing strong positive autocorrelation, suggesting that the data come from an underlying AR process.

**Figure 2.**
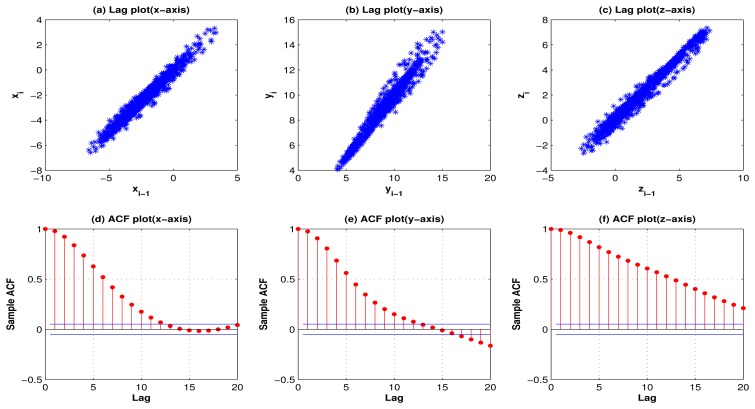
Lag plots (row 1) and autocorrelation plots (row 2) for the 3-axes activity-acceleration signals of walking, showing strong positive autocorrelation, suggesting that the data come from an underlying AR process.

**Figure A1:**
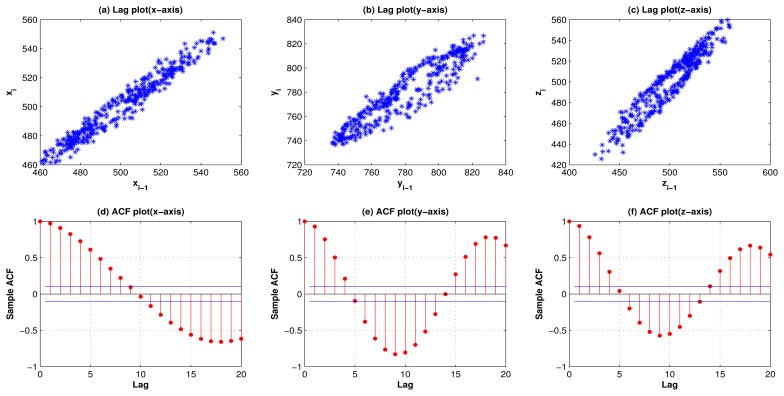
Lag plots (row 1) and autocorrelation plots (row 2) for the 3-axes activity-acceleration signals of upstairs, showing strong positive autocorrelation (with some noise), suggesting that the data come from an underlying AR process.

**Figure A2:**
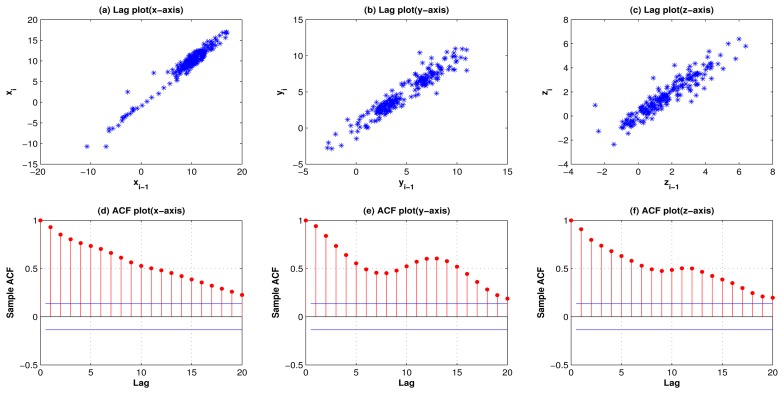
Lag plots (row 1) and autocorrelation plots (row 2) for the 3-axes activity-acceleration signals of downstairs, showing strong positive autocorrelation, suggesting that the data come from an underlying AR process.

**Figure A3:**
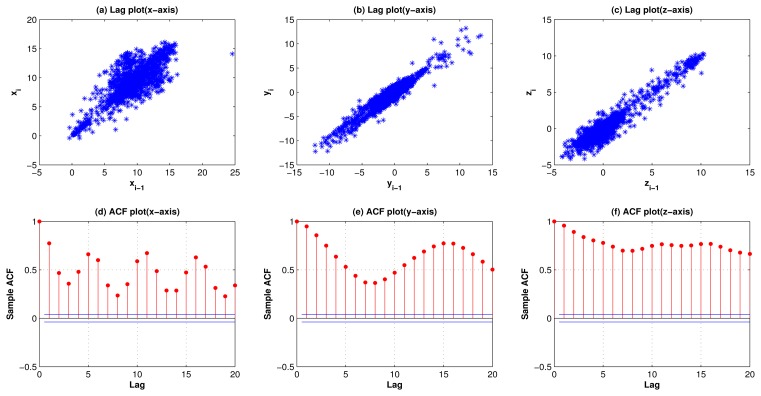
Lag plots (row 1) and autocorrelation plots (row 2) for the 3-axes activity-acceleration signals of running, showing strong positive autocorrelation (except for the x-axis, which shows some noise), suggesting that the data come from an underlying AR process.

**Figure A4:**
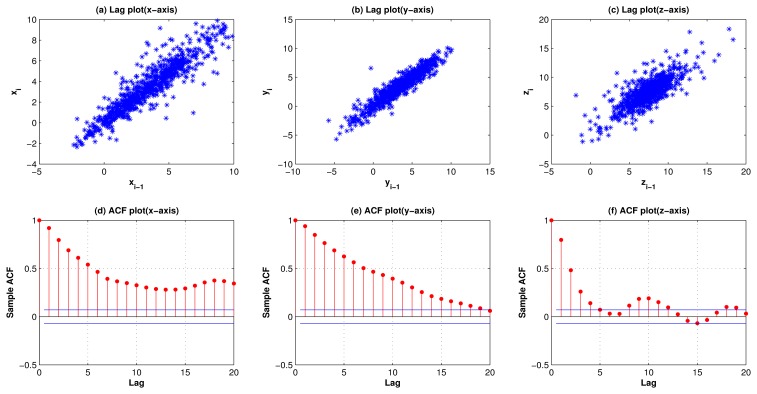
Lag plots (row 1) and autocorrelation plots (row 2) for the 3-axes activity-acceleration signals of hopping, showing strong positive autocorrelation (except for the z-axis, which shows some outliers), suggesting that the data come from an underlying AR process.

Figures 1 and 2 show the lag plots and autocorrelation plots for the 3 axes of standing, and walking. (As for walking-upstairs, walking-downstairs, running, and hopping, please refer to Figures A1, A2, A3 and A4 in the appendix). For the lag plots, see the tight clustering of the data points along the diagonal for almost all the activities. Such a behavior is a signature of a process with strong positive autocorrelation [36]. These processes exhibit highly non-random behavior. In other words, there is a strong association between an observation and a succeeding observation. Meaning, if you know Y*_i_*_−1_ you can make a strong guess as to what *Y_i_* will be. For autocorrelation plots, one can see (in most cases) a high autocorrelation at lag 1 that slowly decreases. The decreasing autocorrelation is generally linear with some noise. Such an autocorrelation plot siginifies the presence of strong autocorrelation in the data [36]. In conclusion, both the lag plots and the autocorrelation plots show strong positive autocorrelation that suggests that the data come from an underlying autoregressive process [37].

### 5.3. Noise Reduction

The lag plots and autocorrelation plots not only suggest that the activity acceleration signals exhibit strong corrleation, but they also show the presence of some outliers (noise). Therefore, to remove this noise a moving average filter of order 3 was employed in this work.

### 5.4. Data Modeling

#### 5.4.1. Autoregressive (AR) Modeling

Since EDA showed that the activity acceleration signals are generated by an AR process, AR models were used to model these signals. AR modeling utilizes the time history of a signal to extract important information hidden in the signal. It is superior to many other methods, especially in biomedical signal processing, as it can take advantage of the noise inherent in a biological system and extract information from propagation of that noise in a signal.

An AR model predicts the current values of a time series from the past values of the same series. Basically, the AR model may be regarded as a set of autocorrelation functions. AR modeling of a time series is based on the assumption that the most recent data points contain more information than the other data points, and that each value of the series can be predicted as a weighted sum of the previous values of the same series plus an error term. The AR model is defined by

(2) $$x[n]=\sum_{i=1}^{M}a_{i}x[n-i]+\varepsilon [n]$$

where *x*[*n*] is the current value of the time series which in our case is the activity acceleration-signal, *a*_1_…*a_M_* are predictor (weighting) coefficients, *M* is the model order that indicates the number of past values used to predict the current value, and *ε*[*n*] represents a one-step prediction error, *i.e.*, the difference between the predicted value and the current value at this point. Further details on AR modeling of a time series can be found in [16].

#### 5.4.2. Model Order and Window Length

There is no straightforward way to determine the correct AR model order. A proper order would yield a good data fitting effect, while retaining a high data compression ratio. In order to determine the optimal AR model order, we adapted the Akaike Information Criteria (AIC) [38].

Although, the root mean square error is generally used to achieve a good estimate of an AR model order, it is still not the most appropriate. AIC is a measure of goodness of fit of an estimated model. Based on the concept of entropy, AIC offers a relative measure of information lost when a given model is used to describe a given time series. Given a dataset, several models are fitted and ranked according to their AIC. The one having the lowest AIC is usually the best model for describing the dataset. AIC is defined by

(3) $$\text{AIC}=-2L_{m}+2_{m}$$

where *L_m_* is the maximized log-likelihood and *m* is the number of parameters in the model. The index takes into account both the statistical goodness of fit and the number of parameters that have to be estimated to achieve this particular degree of fit, by imposing a penalty for increasing the number of parameters. Lower values of the index indicate the preferred model, as the one with the fewest parameters that still provides an adequate fit to the data [38].

**Figure 3.**
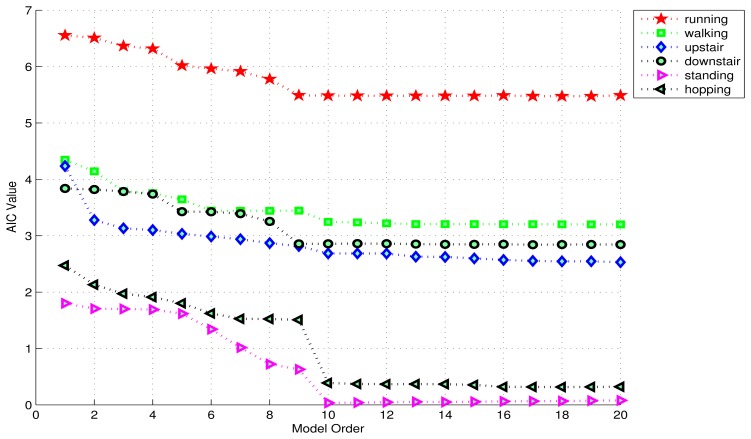
The average AIC values for 3-axes plotted against model order for the 6 activities. AIC curves for all activities tend to even out near 10, suggesting that 10 is the appropriate model order in this case.

Before computing AIC for different orders, one must choose the window-length. Since the most appropriate window-length is not known at this point, AIC values for different orders were computed using a window-length of 1 s. Figure 3 shows the AIC plots for the 6 activities. Each AIC value in these plots represents an average of the AIC values for the 3 axes. It can be seen that the AIC curves for almost all the activities even out near the model order 10, *i.e.*, 10 coefficients per axis, suggesting that 10 is the most appropriate model order in this case.

**Figure 4.**
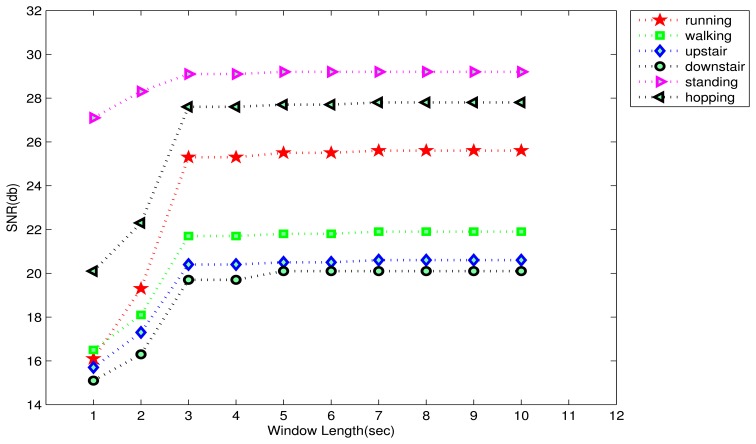
The average SNR values for 3 axes plotted against window-length for the 6 activities. SNR curves for all activities tend to even out near the window-size of 3 s (10 per axis) suggesting that it is the appropriate window-length in this case.

The criteria used for selecting the most suitable window-length for the chosen model order, in this work, is signal-to-noise ratio (SNR). For each activity, an AR-model of order 10 was fitted for windows of different length (shortest window: 1 s, longest window: 10 s) for each axis. In each case, the model was then used to generate a simulated signal and the SNR was computed for both the original and the simulated signals as following:

(4) $$\text{SNR}=10\log\frac{\sum_{i=1}^{N}{\left(\upsilon (i)\right)}^{2}}{\sum_{i=1}^{N}{\left(\upsilon (i)-\bar{\upsilon }(i)\right)}^{2}}$$

where *ν*(*i*) and *ν̄*(*i*) are the original and the simulated signal at the *i*-th instant, and *N* is the length of the modeled signal. The average SNR values for 3 axes, for 6 activities, and for different windows are summarized in Figure 4. It can be seen that the SNR curves for almost all the activities even out near the window-size of 3 s. After this point, no significant gain in the SNR values were obtained. Thus, it can be easily concluded that the window-size of 3 s, *i.e.*, 60 samples per axis is the most appropriate, as it offers the same goodness of fit as larger windows, and it is not too long to result in a delayed-response, which is desirable considering the real-time requirements of a smartphone-based HAR system.

#### 5.4.3. Model Validation

Once the model has been identified, its validity must be checked. The primary tool for model diagnostic checking is the analysis of the residuals, *i.e.*, the prediction error sequence. If the chosen model is a good model for the data, the residuals should be white noise, drawn from a fixed distribution with a constant mean and variance [37]. To validate whether the selected AR model of order 10, given a window-length of 3 s, is a good model, this validation criteria was employed.

To validate the chosen model, the AR coefficients were estimated for each activity for a subject using a certain sample. These parameters were then used to create copies of the modeled signals, which were compared with the original data for the same activity from the same subject but from a different sample to collect the residuals. Different samples were chosen to analyze how well a model estimated using a certain sample can be used to fit data from a different sample.

**Figure 5.**
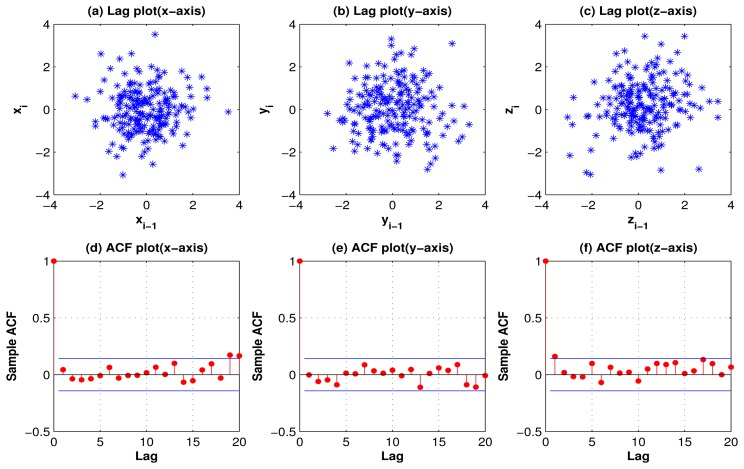
Lag plots (row 1) and autocorrelation plots (row 2) for the 3-axes residuals of walking show no structure or autocorrelation, suggesting that the data are white noise.

Figure 5 shows the 3-axes lag plots and the autocorrelation plots for the residuals of walking for 1 subject. Both the lag plots and autocorrelation plots show that the data are random as they exhibit no structure or correlation. In other words, one cannot infer the next value *Y_i_* from a current value *Y_i_*_-1_. This indicates that the chosen AR model of order 10 is a good model.

### 5.5. Kernel Discriminant Analysis (KDA)

**Figure 6.**
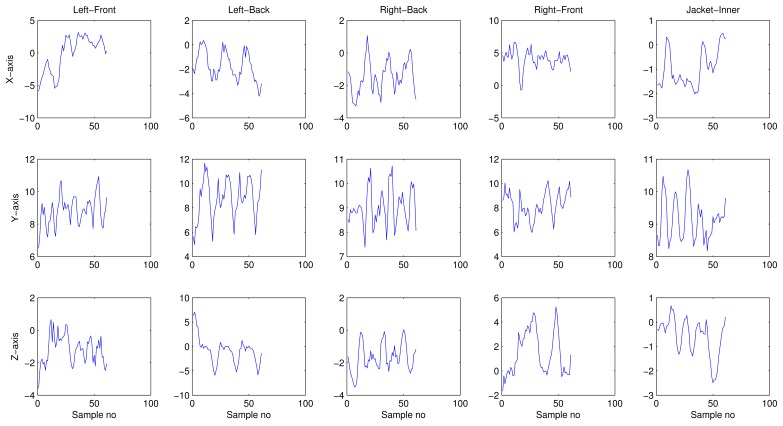
Activity acceleration signals for walking from 5 different positions.

An accelerometers output is very sensitive to the position of the human body in which it is placed. Figure 6 shows the activity acceleration signals for 5 phone-positions (trousers' front pockets, trousers' back pockets, and jacket's inner pocket). These signals were captured while a subject walked along an L-shaped corridor, carrying 5 phones (1 in each position). It clearly shows how different the output of a smartphone's accelerometer can be, for the same activity, when carried in different positions. Such differences result in high within-class variance, which could lead to low classification accuracy. Meaning, though AR coefficients show promise in serving as light-weight and efficient features for smartphone-based HAR, the presence of high within-class variance limits their application. Therefore, KDA was used to overcome this problem.

KDA is a non-linear discriminating approach, which seeks non-linear discriminating features using kernel techniques. Suppose we have a set of *m* feature vectors x_1_, x_2_, ⋯, x*_m_* ∈ R^3^*^p^* belonging to *C* activity classes where *p* is the AR-model order. Let

(5) $${\text{x}}_{i}={[}^{a_{x1,}a_{x2,}\cdots ,a_{xp,}a_{y1,}a_{y2,}\cdots ,a_{yp,}a_{z1,}a_{z2,}\cdots ,a_{zp}]T}$$

where *a_xi_*, *a_yi_*, and *a_zi_* are the AR coefficients for 3 axes. We considered the problem in a feature space *F* induced by some non-linear mapping *φ* : R^3^*^p^* → *F*. *φ* was chosen to be the radial basis function. For a properly chosen φ, an inner product 〈,〉 can be defined in *F* which makes for the so called reproducing the kernel Hilbert space. More specifically, 〈*φ*(x*_i_*),*φ*(x*_j_*)〉 = K (x*_i_*, x*_j_*) holds where *K* (., .) is a positive semi-definite kernel function. To find the linear discriminant in *F*, the following criterion needs to be maximized

(6) $$J(\omega )=\frac{\omega^{T}S_{b}^{\varphi }\omega }{\omega^{T}S_{\omega }^{\varphi }\omega }$$

where *ω* is the KDA basis vector, $S_{b}^{\varphi }$ and $S_{\omega }^{\varphi }$ are the between-class and within-class scatter matrices in *F*, and are defined as

(7) $$S_{b}^{\varphi }=\sum_{k=1}^{C}m_{k}(\mu_{\varphi }^{k}-\mu_{\varphi }){\left(\mu_{\varphi }^{k}-\mu_{\varphi }\right)}^{T}$$

(8) $$S_{\omega }^{\varphi }=\sum_{k=1}^{C}\left(\sum_{i=1}^{m_{k}}(\varphi (x_{i}^{k})-\mu_{\varphi }^{k}){\left(\varphi (x_{i}^{k})-\mu_{\varphi }^{k}\right)}^{T}\right)$$

where $\mu_{\varphi }^{k}$ and *μ_φ_* are the mean of the *k*-th class and the global mean, respectively. *m_k_* is the number of samples in the *k*-th class. The solution to Equation (6) is a linear combination of *φ*(x*_i_*) with coefficients *α_i_*; such that

(9) $$\omega =\sum_{i=1}^{m}\alpha_{i}\varphi (x_{i})$$

Let *α* = [*α*_1_, ⋯, *α_m_*]*^T^*, and it can be proved that Equation (4) is equivalent to

(10) $$J(\alpha )=\frac{\alpha^{T}\text{KWK}\alpha }{\alpha^{T}\text{KK}\alpha }$$

and the optimal *α*(s) are given by the eigen vectors with respect to the maximum eigen values of

(11) KWKα=λKKα

where **K** is the kernel matrix (**K***_ij_*= K(x*_i_*, x*_j_*)) and **W** is defined as

(12) $${\text{W}}_{ij}=\{\begin{array}{cc} 1/m_{k}, & \text{if}\;{\text{x}}_{i}\;\text{and}\;{\text{x}}_{j}\;\text{belong to}\;k-\text{th class}\\ 0, & \text{otherwise} \end{array}$$

For a new pattern x, its projection onto a KDA basis vector *ω* in *F* is calculated as

(13) $$\left(\omega ,\varphi (\text{x}))=\alpha^{T}\text{K}(:,\text{x}\right)$$

where

(14) $$\text{K}(:,\text{x})={\left[\text{K}({\text{x}}_{1},\text{x}),\cdots ,\text{K}({\text{x}}_{m},\text{x})\right]}^{T}$$

For more details on KDA please refer to [39]

### 5.6. Classifier

As for the classifier, we decided to use the standard, feed-forward, and backpropagation artificial neural networks (ANNs) based on the findings of our previous study [12]. In that study, the performance of several automatic classification methods, including decision trees [40] nearest neighbor and Bayesian Networks [41] and ANNs [42], were compared. Finally, the ANNs were selected for their better performance.

## 6. Experimental Results

The performance of using the AR coefficients as features for real-time/online activity recognition was compared against 3 feature extraction methods: FFT and DCT coefficients (the 2 most commonly used frequency domain features in traditional wearable accelerometer based AR systems), and a 42-dimensional time domain (TD) feature used in [27] (a smartphone based AR system).

These comparisons were performed using a subject-independent recognition test. Under this setting, 10 new subjects (6 males and 4 females) were recruited. These subjects belonged to different age groups: 2 subjects had the same age group as the subjects who collected the training data, 2 were aged between 18–20 years old, whereas the rest of the 6 subjects were between the ages of 45–50 years old. Moreover, they also had different physical characteristics, i.e., height, weight, and built. The goal was to have our models trained using data from a certain age group, and have them tested for subjects from different age groups, and with different physical characteristics. The subjects carried phones with a custom-built Android application for capturing the acceleration data, computing the features, classifying the activity, and storing the true label, as well as the classified label, in a database.

Burg's method [43], also called maximum entropy method, was implemented in Java for computing the AR coefficients. FFT features were computed using FFTW [44], which is a C subroutine library for computing the discrete Fourier transform (DFT). As for the DCT, we implemented the algorithm of Fast Discrete Cosine Transform (FDCT) in Java. For TD features, a Java program was implemented following the instructions provided in [27]. During training, features were computed offline using the dataset described in Section 4. The same window-length, *i.e.*, 3 s was used for computing each feature set.

KDA was implemented in Java, and applied to each feature set, and the resulting features were used to train the ANNs. The ANNs were implemented using Neuroph, which is a Java neural network framework. There were 24 ANNs in total: 6 ANNs, corresponding to 6 different sampling rates, for the 4 feature extraction methods. Each network had n input neurons (where *n* corresponds to the dimentions of the feature vector after KDA), 1 hidden layer with 3 neurons, and 6 output neurons corresponding to the 6 activities. A different number of hidden layers and neurons was tested for each ANN to optimize the accuracy, and at the end, the given settings were chosen. Once trained, the ANNs were transferred to the SD cards. Following 4 experiments were then performed to evaluate the proposed system.

### 6.1. First Experiment

In this experiment, each subject performed the same activities in a random order in 5 different sessions over a period of one week. Each session was 20 min long. During this experiment, the phones were configured to provide data at the sampling rate of 20 Hz. Four ANNs trained with KDA features, for the 4 feature extraction methods, using the 20 Hz training-data were used for classification. The classification results were stored in a database. Finally, the recognition accuracy was evaluated by comparing the recognized lables for the activities with their true lables. The recognition results for FFT, DCT, TD, and AR-features are summarized in Tables 1, 2, 3 and 4, with an average accuracy of 45.41%, 48.55%, 67.58%, and 87.1%, respectively.

### 6.2. Second Experiment

**Figure 7.**
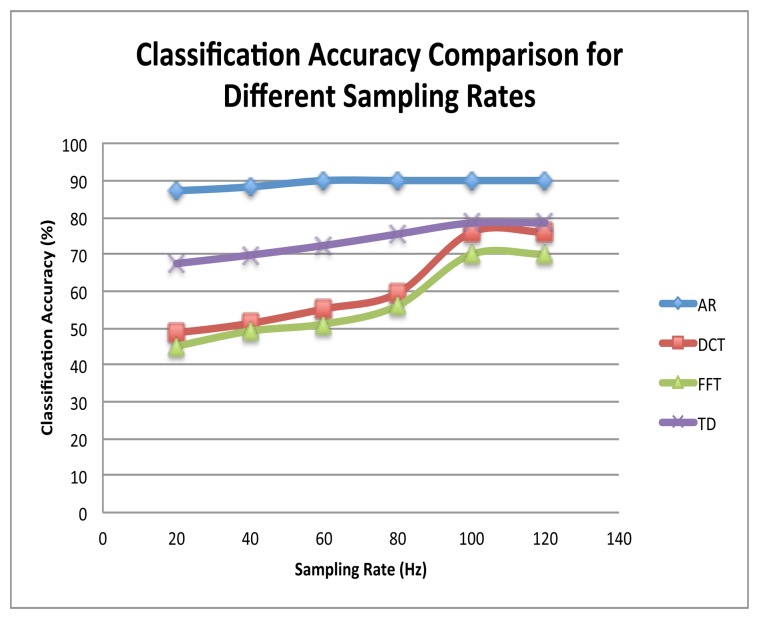
A comparison of the classification accuracies of AR, FFT, and DCT features for different sampling rates. It can be seen that the AR-coefficients provided almost the same accuracy for small and large sampling rates, whereas the other 3 needed a sampling rate of almost 100 Hz (about 5-times the sampling rate for AR-features) to achieve a close accuracy.

In this experiment, we studied the effect of increasing the sampling rate on the recognition accuracy of each feature. The same 10 subjects performed 5 evaluation sessions (each 20 min long) with sampling rates of 40 Hz, 60 Hz, 80 Hz, 100 Hz, and 120 Hz, respectively. In each evaluation session, the ANNs corresponding to the used sampling rate were used for classification. It should be noted that during the first experiment, to get a fair comparision, first 10 FFT and DCT features were extracted from each axis of the acceleration data before the application of KDA (just like the AR coefficients). However, for this experiment, different number of features were extracted for both FFT and DCT, *i.e.*, 40 per axis for FFT and 48 per axis for DCT, as per the findings of [31,32], respectively. The number of TD features, on the other hand, was kept constant in each experiment, *i.e.*, 42. The classification results for this experiment are summarized in Figure 7.

### 6.3. Third Experiment

**Figure 8.**
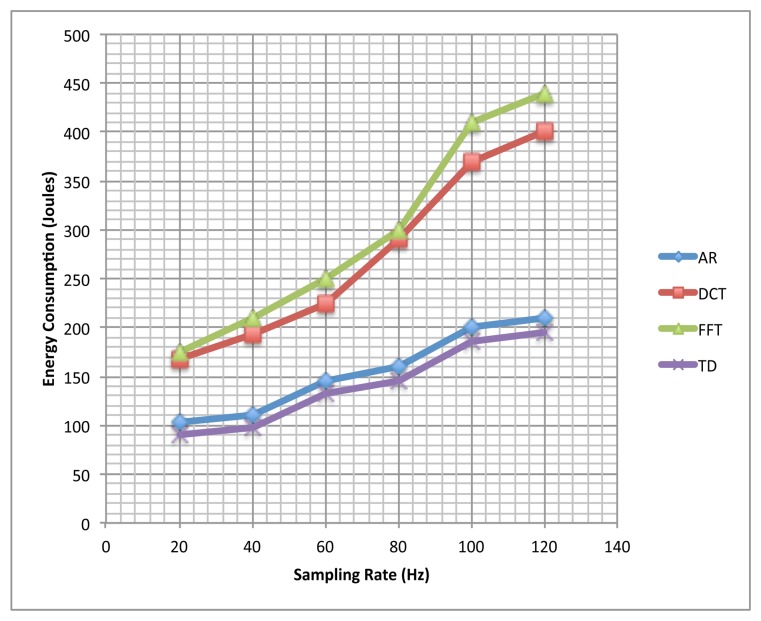
Energy consumption for LG Nexus 4 for different sampling rates and feature extraction methods.

In this experiment, we calculated the the energy consumption (in Joules) for different sampling rates and features, for our representative phone: LG Nexus 4. The results are summarized in Figure 8. The network interfaces and the display were powered off while obtaining these readings.

### 6.4. Fourth Experiment

As mentioned earlier, in this work KDA was used to overcome the with-in class variability caused by placing the phones at different positions. In this experiment, we evaluated the advantage of using this method. The same 10 subjects performed 5 evaluation sessions, corresponding to 5 positions. Each session was 20 min long. Their phones were equipped with 2 ANNs: the first ANN was trained with AR features (without KDA), whereas the second ANN was trained with KDA features that were obtained by applying KDA on AR features. The sampling rate was 20 Hz. In all 5-evaluation sessions, the subjects performed the same routine that consisted of a random sequence of the 6 activities, while choosing a different position each time. The average recognition accuracies for different positions for all activities, and for all subjects (with and without KDA) are summarized in Table 5, which prove the advantage of using this method.

## 7. Discussions

It can be seen that the recognition rates for FFT, DCT, and TD features were low as compared to AR features in the first experiment (the base case, with a sampling rate of 20 Hz). It does not mean that these methods are not suitable for accelerometer-sensor based HAR. High recognition rates have been achieved in the past using these methods when coupled with high sampling rates and large data windows [27,31,32]. The findings of the second experiment support this fact, where the classification accuracy using the AR-features changed by just 3% with the increase in the sampling rate; however, the accuracy of FFT, DCT, and TD features improved significantly, and a comparable accuracy (though still lower than that of the AR-features) was achieved with high sampling rates. However, such settings are not suitable for smartphone-based HAR systems, as employing high sampling rates increases energy consumption (as shown in Figure 8), thereby reducing the battery life.

We can easily make the following observations from Figure 8: (1) The energy overhead in continuous activity recognition on smartphones clearly increases with sampling rate; (2) It is less expensive to utilize AR features, at almost all the sampling rates, as opposed to DCT and FFT features; (3) The energy overhead of using TD features is almost the same as that of AR features; however, to achieve a good recognition accuracy with TD features one must use a high sampling rate, coupled with long data windows, which makes them more expensive than AR features. Thus the intensity and frequency characteristics of activity acceleration signals, use of small time windows (3 s), low sampling rate (20 Hz), and high recognition accuracy show the feasibility of using the AR coefficients as features for subject-independent real-time smartphone-based HAR.

It should be noted that the training of ANN and the calculations of KDA basis vectors were done offline, which were then transferred to the mobile device. During online recognition on the device, KDA basis vectors and ANNs were simply used for data projection and classification, respectively. This process is significantly light-weight as compared to the training phase, which happens offline. It is the feature extraction that always happens on the device, and that is why this study focused on finding light-weight and accurate features for online activity recognition on the phone.

When compared with some existing techniques, the recognition accuracy in this work (87.1%) appears to be a bit lower. For example, [27] achieved an accuracy of 95.8% and 93.9% using quadratic discriminant analysis and k-nearest neighbors, respectively. However, that is not the case. This difference in accuracies can be attributed to 4 factors. Firstly, they employed a sampling rate of 40 Hz, which is twice as much as the one used in our base case, *i.e.*, 20 Hz. Secondly, the features in their work were computed using a data-window of 7.5 s which is twice as big as the one used in this work, *i.e.*, 3 s. Thirdly, they used only pants' front pockets to hold the device, whereas in our case, subjects were allowed to carry their phones in 3 different positions. It should be noted that 5 out of 6 activities considered in this work involve movement of the legs and choosing a pocket far from the legs (such as a jacket's inner pocket) can alter the output of the acceleration signal significantly, thereby introducing a large within-class variance that can lead to a high number of misclassification. Finally, they used 7 subjects for online evaluation of their system, and only 3 of these subjects were those whose data were not used to train the recognition model. On the other hand, in this study, online evaluation was performed using 10 subjects none of who took part in training data collection. Moreover, these subjects belonged to 3 different age groups, and had different physical characteristics. Given all these factors, an accuracy of 87.1% appears to be very reasonable, especially given a low sampling rate of 20 Hz and a small data-window of 3 s.

Though the implemented system works for different positions, it is still not ideal for a real-life scenario as in real-life users can carry the phones anywhere while performing an activity, such as holding the device in the hands, in a bag, or in a jacket's side pockets, etc. Implementing such a system requires features that are position/orientation independent. One possible solution could be to design a standard/global reference coordinate space, project the original data (which might be coming from different positions, each with its own local coordinate system) onto this reference space for the sake of standardization, and then continue with feature extraction. However, this is just a hypothesis whose evaluations require time. It will also need a large amount of acceleration data collected from various different positions using a large number of subjects. Therefore, we plan to examine this hypothesis in future studies.

Finally, though the system provides reasonable accuracy for subject-independent recognition, it is still desirable to find ways to increase this accuracy further. One possibility could be to use the data collected during the usage-state (when a phone with a trained classifier is being used by a new user for online recognition) to improve the recognition rate for the new user. However, implementation of such approaches becomes challenging in terms of computational complexity, and we plan to investigate this issue in our future work.

## 8. Conclusions

This paper aims to show the suitability of using AR modeling as a feature extraction technique for real-time subject-independent human activity recognition on a smartphone with a built-in accelerometer. Acceleration data for 6 activities collected from multiple subjects, of different gender and age, were analyzed to show that these signals are generated by an AR process, which makes AR coefficients a suitable choice in representing them in the feature space.

It is shown that not only these models are robust in describing these signals across multiple subjects; they do not require longer time windows and high sampling rate for their computation, too. This helps in achieving fast/real-time response and preserving phone's battery life. Moreover, the classification results for our real-time subject-independent classification (using the subjects which were not part of the training data) show that the chosen features outperformed the other commonly used methods by up to 40% in terms of accuracy for a sampling rate and a window-size of 20 Hz and 3 s, respectively.

## Figures and Tables

**Table 1.** The Confusion Matrix for ANN (20 Hz) with FFT-features (Unit: %).

**Activity**	**Standing**	**Walking**	**Running**	**Upstairs**	**Downstairs**	**Hopping**
Standing	**40.5**	12.6	7.5	12	13.4	11
Walking	9.6	**44.4**	13	9.5	12.5	12
Running	4.4	14.6	**45**	11.3	10.1	14.6
Upstairs	4	10.2	14	**51**	10.8	10
Downstairs	12.3	17.7	10	9.5	**40.5**	10
Hopping	7.5	10	9.3	9.5	12.7	**51**

Mean			45.41			

**Table 2.** The Confusion Matrix for ANN (20 Hz) with DCT-features (Unit: %).

**Activity**	**Standing**	**Walking**	**Running**	**Upstairs**	**Downstairs**	**Hopping**
Standing	**49.5**	10.6	5.5	9.2	12.6	12.6
Walking	8.5	**46.5**	10	9.5	12.5	13
Running	4	12.6	**50**	10.1	7.9	15.4
Upstairs	4	10.2	11	**55.8**	9	10
Downstairs	9	20	10	9	**41.5**	10.5
Hopping	6	10.5	12	10.5	13	**48**

Mean			48.55			

**Table 3.** The Confusion Matrix for ANN (20 Hz) with TD-features (Unit: %).

**Activity**	**Standing**	**Walking**	**Running**	**Upstairs**	**Downstairs**	**Hopping**
Standing	**94**	2	0	1	1	2
Walking	2	**56.5**	5.5	14	15	7
Running	2	8	**70**	7	7	6
Upstairs	3	14	5	**58**	15	5
Downstairs	2	16	5	14	**57**	6
Hopping	2	6	9	6	7	**70**

Mean			67.58			

**Table 4.** The Confusion Matrix for ANN (20 Hz) with AR-features (Unit: %).

**Activity**	**Standing**	**Walking**	**Running**	**Upstairs**	**Downstairs**	**Hopping**
Standing	**95**	1	0	0	4	0
Walking	1	**86.1**	3	1	6	2.9
Running	0	2	**90**	3	0	5
Upstairs	0	3.5	5	**83.5**	3	5
Downstairs	4	5.5	3	2	**82.5**	3
Hopping	2	2	5	2	3	**86**

Mean			87.1			

**Table 5.** The overall average recognition accuracies for 5 positions for all activities, and for all subjects, using AR features at 20 Hz with and without KDA (Unit: %).

**Position**	**With KDA**	**Without KDA**
Pants' front pocket (left)	87.1	74
Pants' front pocket (right)	87.3	74.2
Pants' back pocket (left)	87.2	73.6
Pants' back pocket (right)	87	73.1
Jacket's inner pocket	86.3	68.2
Mean	86.98	72.62

## References

[1] Tolstikov A., Hong X., Biswas J., Nugent C., Chen L., Parente G. (2011). Comparison of fusion methods based on DST and DBN in human activity recognition. J. Control Theory Appl..

[2] Yang J., Lee J., Choi J. (2011). Activity Recognition based on RFID object usage for smart mobile devices. J. Comput. Sci. Technol..

[3] Sarkar J., Vinh L., Lee Y.K., Lee S. (2011). GPARS: A general-purpose activity recognition system. Appl. Intell..

[4] Ribeiro P., Santos-Victor J. Human Activity Recognition from Video: Modeling, Feature Selection and Classification Architecture.

[5] Candamo J., Shreve M., Goldgof D., Sapper D., Kasturi R. (2010). Understanding transit scenes: A survey on human behavior-recognition algorithms. IEEE Trans. Intell. Transp. Syst..

[6] Mathie M., Coster A., Lovell N., Celler B. (2004). Accelerometry: Providing an integrated, practical method for long-term, ambulatory monitoring of human movement. Physiol. Meas..

[7] Najafi B., Aminian K., Paraschiv-Ionescu A., Loew F., Bula C., Robert P. (2003). Ambulatory system for human motion analysis using a kinematic sensor: Monitoring of daily physical activity in the elderly. IEEE Trans. Biomed. Eng..

[8] Bao L., Intille S. (2004). Activity Recognition from User-Annotated Acceleration Data.

[9] Ravi N., Dandekar N., Mysore P., Littman M. Activity Recognition from Accelerometer Data.

[10] Wang S., Yang J., Chen N., Chen X., Zhang Q. Human Activity Recognition with User-Free Accelerometers in the Sensor Networks.

[11] Mathie M., Coster A., Lovell N., Celler B., Lord S., Tiedemann A. (2004). A pilot study of long-term monitoring of human movements in the home using accelerometry. J. Telemed. Telecare.

[12] Khan A., Lee Y., Kim T. Accelerometer Signal-Based Human Activity Recognition Using Augmented Autoregressive Model Coefficients and Artificial Neural Nets.

[13] Huynh T., Schiele B. Analyzing Features for Activity Recognition.

[14] Kao T.P., Lin C.W., Wang J.S. Development of a Portable Activity Detector for Daily Activity Recognition.

[15] Jatobá L.C., Grossmann U., Kunze C., Ottenbacher J., Stork W. Context-Aware Mobile Health Monitoring: Evaluation of Different Pattern Recognition Methods for Classification of Physical Activity.

[16] Helmi M., AlModarresi S. Human Activity Recognition Using a Fuzzy Inference System.

[17] Zhang M., Sawchuk A.A. A Bag-of-Features-Based Framework for Human Activity Representation and Recognition.

[18] Casale P., Pujol O., Radeva P. (2011). Human activity recognition from accelerometer data using a wearable device. Pattern Recognit. Image Anal..

[19] Fahim M., Fatima I., Lee S., Park Y.T. (2013). EFM: Evolutionary fuzzy model for dynamic activities recognition using a smartphone accelerometer. Appl. Intell..

[20] Lara O.D., Labrador M.A. A Mobile Platform for Real-Time Human Activity Recognition.

[21] Berchtold M., Budde M., Gordon D., Schmidtke H., Beigl M. Actiserv: Activity Recognition Service for Mobile Phones.

[22] Kwapisz J.R., Weiss G.M., Moore S.A. (2011). Activity recognition using cell phone accelerometers. ACM SIGKDD Explor. Newsl..

[23] Győrbíró N., Fábián Á., Hományi G. (2009). An activity recognition system for mobile phones. Mob. Netw. Appl..

[24] Lu H., Yang J., Liu Z., Lane N.D., Choudhury T., Campbell A.T. The Jigsaw Continuous Sensing Engine for Mobile Phone Applications.

[25] Sun L., Zhang D., Li B., Guo B., Li S. Activity Recognition on an Accelerometer Embedded Mobile Phone with Varying Positions and Orientations.

[26] Wang S., Chen C., Ma J. Accelerometer Based Transportation Mode Recognition on Mobile Phones.

[27] Siirtola P., Rö ning J. (2012). Recognizing human activities user-independently on smartphones based on accelerometer data. Int. J. Interact. Multimed. Artif. Intell..

[28] Frank J., Mannor S., Precup D. (2011). Activity recognition with mobile phones. Mach. Learn. Knowl. Dis. Databases.

[29] Gomes J.B., Krishnaswamy S., Gaber M.M., Sousa P.A.C., Menasalvas E. MARS: A Personalised Mobile Activity Recognition System.

[30] Chen Y.P., Yang J.Y., Liou S.N., Lee G.Y., Wang J.S. (2008). Online classifier construction algorithm for human activity detection using a tri-axial accelerometer. Appl. Math. Comput..

[31] Mantyjarvi J., Lindholm M., Vildjiounaite E., Makela S.M., Ailisto H. Identifying Users of Portable Devices from Gait Pattern with Accelerometers.

[32] He Z., Jin L. Activity Recognition from Acceleration Data Based on Discrete Consine Transform and SVM.

[33] Khan A.M., Lee Y.K., Lee S.Y., Kim T.S. (2010). A triaxial accelerometer-based physical-activity recognition via augmented-signal features and a hierarchical recognizer. IEEE Trans. Inf. Technol. Biomed..

[34] Ryder J., Longstaff B., Reddy S., Estrin D. Ambulation: A Tool for Monitoring Mobility Patterns over Time Using Mobile Phones.

[35] Zhang S. (2012). Smartphone Based Activity Recognition System. Ph.D. Thesis.

[36] Natrella M., Croarkin C. (2010). NIST/SEMATECH e-Handbook of Statistical Methods.

[37] Takalo R., Hytti H., Ihalainen H. (2005). Tutorial on univariate autoregressive spectral analysis. J. Clin. Monit. Comput..

[38] Akaike H. (1974). A new look at the statistical model identification. IEEE Trans. Autom. Control.

[39] Baudat G., Anour F. (2000). Generalized discriminant analysis using a kernel approach. Neural Comput..

[40] Ermes M., Parkka J., Mantyjarvi J., Korhonen I. (2008). Detection of daily activities and sports with wearable sensors in controlled and uncontrolled conditions. IEEE Trans. Inf. Technol. Biomed..

[41] Asada H.H., Shaltis P., Reisner A., Rhee S., Hutchinson R.C. (2003). Mobile monitoring with wearable photoplethysmographic biosensors. IEEE Eng. Med. Biol. Mag..

[42] Foerster F., Smeja M., Fahrenberg J. (1999). Detection of posture and motion by accelerometry: A validation study in ambulatory monitoring. Comput. Hum. Behav..

[43] Burg J.P. Maximum Entropy Spectral Analysis.

[44] Frigo M., Johnson S.G. (2005). The design and implementation of FFTW3. IEEE Proc..

